# Epicardial adipose tissue volume and coronary calcification among people living with diabetes: a cross-sectional study

**DOI:** 10.1186/s12933-021-01225-6

**Published:** 2021-02-05

**Authors:** Emmanuel Cosson, Minh Tuan Nguyen, Imen Rezgani, Sopio Tatulashvili, Meriem Sal, Narimane Berkane, Lucie Allard, Pierre-Yves Brillet, Hélène Bihan

**Affiliations:** 1 Department of Endocrinology-Diabetology-Nutrition, AP-HP, Avicenne Hospital, Université Paris 13, Sorbonne Paris Cité, CRNH-IdF, CINFO, Bobigny, France; 2 Unité de Recherche Epidémiologique Nutritionnelle, UMR U1153 INSERM/U11125 INRA/CNAM/Université Paris 13, Bobigny, France; 3 Department of Functional Explorations, AP-HP, Jean Verdier Hospital, Université Paris 13, Bondy, France; 4 Department of Radiology, AP-HP, Avicenne Hospital, Bobigny, France; 5 Laboratoire Educations et Pratiques de Santé UR 3412, UFR Santé, Médecine, Biologie Humaine, Université Paris Sorbonne Paris Nord, 74, rue Marcel Cachin, 93017 Bobigny Cedex, France

**Keywords:** Computed tomography, Coronary artery calcification, Diabetes, Epicardial adipose tissue, Epicardial fat tissue, Metabolic syndrome, Visceral fat

## Abstract

**Background:**

Epicardial adipose tissue (EAT) has anatomic and functional proximity to the heart and is considered a novel diagnostic marker and therapeutic target in cardiometabolic diseases. The aim of this study was to evaluate whether EAT volume was associated with coronary artery calcification (CAC) in people living with diabetes, independently of confounding factors.

**Methods:**

We included all consecutive patients with diabetes whose EAT volume and CAC score were measured using computed tomography between January 1, 2019 and September 30, 2020 in the Department of Diabetology-Endocrinology-Nutrition at Avicenne Hospital, France. Determinants of EAT volume and a CAC score ≥ 100 Agatston units (AU) were evaluated.

**Results:**

The study population comprised 409 patients (218 men). Mean (± standard deviation) age was 57 ± 12 years, and 318, 56 and 35 had type 2 (T2D), type 1 (T1D), or another type of diabetes, respectively. Mean body mass index (BMI) was 29 ± 6 kg/m^2^, mean AET volume 93 ± 38 cm^3^. EAT volume was positively correlated with age, BMI, pack-year smoking history and triglyceridaemia, but negatively correlated with HDL-cholesterol level. Furthermore, it was lower in people with retinopathy, but higher in men, in Caucasian people, in patients on antihypertensive and lipid-lowering medication, in people with nephropathy, and finally in individuals with a CAC ≥ 100 AU (CAC < 100 vs CAC ≥ 100: 89 ± 35 vs 109 ± 41 cm^3^, respectively, p < 0.05). In addition to EAT volume, other determinants of CAC ≥ 100 AU (n = 89, 22%) were age, T2D, ethnicity, antihypertensive and lipid-lowering medication, cumulative tobacco consumption, retinopathy, macular edema and macrovascular disease. Multivariable analysis considering all these determinants as well as gender and BMI showed that EAT volume was independently associated with CAC ≥ 100 AU (per 10 cm^3^ increase: OR 1.11 [1.02–1.20]).

**Conclusions:**

EAT volume was independently associated with CAC. As it may play a role in coronary atherosclerosis in patients with diabetes, reducing EAT volume through physical exercise, improved diet and pharmaceutical interventions may improve future cardiovascular risk outcomes in this population.

## Background

Type 1 (T1D) and type 2 diabetes (T2D) are associated with an increased risk of cardiovascular disease, irrespective of improved multifactorial care [1, 2]. Epicardial adipose tissue (EAT) has recently been proposed as one determinant contributing to the pathophysiology of cardiovascular complications [3–6]. A recent meta-analysis showed that individuals with diabetes had higher EAT volumes than healthy controls, irrespective of T1D or T2D status and the method used to quantify EAT volume [3].

EAT is located between the myocardium and the visceral pericardium and is considered the heart’s visceral adipose tissue [3–6]. It secretes inflammatory factors and lipid metabolites, and has been suggested as a possible determinant of accelerated atherosclerosis [3–6]. However, at the clinical level, evidence that EAT is a marker of subclinical atherosclerosis in diabetic populations is still limited [5].

Coronary artery calcium (CAC) is considered a good marker of coronary risk [7]. The CAC score assesses the volume of coronary calcifications located in atherosclerotic plaques and provides a summary measure of atherosclerotic disease, reflecting the cumulative lifetime effect of risk factors and genetic and environmental factors [7]. The CAC score increases with cardiovascular risk in both the general population and in people with diabetes [8–10] and a threshold of 100 Agatston units (AU) is commonly used to identify the individuals at high risk [7–10]. To date, only four studies which have evaluated the association either between EAT volume [11–13] or EAT thickness [14] and CAC score in patients with diabetes have had mixed results: one study showed a positive association between EAT and CAC score in 333 patients without cardiovascular disease or renal impairment [11]; another only showed an association in the 38 individuals out of 95 studied who had early-onset T2D [13], while the remaining two studies reported no association whatsoever [12, 14].

In this context, the present study aimed to evaluate, in a large cohort of people living with diabetes, whether EAT volume was associated with CAC score, independently of confounding factors.

## Methods

### Study population

The present retrospective study involved each consecutive patient with diabetes admitted to the Department of Diabetology-Endocrinology-Nutrition, in Avicenne Hospital, Bobigny, France, between January 1, 2019 and September 30, 2020. All had a computed tomography (CT) scan during hospitalization to evaluate their CAC score (AU) and assess their cardiovascular risk [8, 10].

### CT imaging

CAC scores and EAT volume were calculated using ECG-gated cardiac CT without contrast injection. All CT scans were performed with GE (Healthcare Digital, France) or Siemens (Healthineers, France) scanners. CAC scores were calculated according to guidelines [15] using the dedicated tool available on Picture Archiving and Communication Systems (PACS) platforms (either from Carestream Health, Rochester, NY or Philips Healthcare, Best, the Netherlands). EAT volume was quantified with the software package AW VolumeShare 7 (GE Healthcare Digital). It was measured using a semi-automatic segmentation technique on every axial slice from the thoracic inlet to the beginning of the abdomen. The software automatically measured EAT volume (in cm^3^) by summing appropriate pixels using a CT Hounsfield unit, range − 150 to − 50 HU.

### Data collection

Data were extracted from patients’ medical records and collected in a secure health database. For the present study, we focused on:

- General data: current tobacco consumption and pack-year smoking history, diagnosed premature (before 55 years for men; before 65 years for women) coronary artery disease in first degree relatives; ethnicity (recorded as Caucasian, Arabic (Middle East, North Africa), Afro-Caribbean (African, African American, Caribbean), Asian (Asian continent), or other).
- Medical history: routine treatments before admission, history of stroke, heart failure, or coronary artery disease. Hypertension and dyslipidaemia were self-reported and/or inferred from blood pressure- and lipid-lowering agents, respectively. Additionally, we collected data to measure possible overweightness (body mass index (BMI) ≥ 25 kg/m^2^) and obesity (BMI ≥ 30 kg/m^2^). BMI was calculated using the formula: weight (kg)/height^2^ (m^2^). Weight and height were measured within 24 h of hospital admission.
- Biomarkers: HbA1c (high performance liquid chromatography variant); total and HDL cholesterol (colorimetric assay on homogenous phase and cholesterol dosage by cholesterol oxidase), triglycerides (colorimetric assay), and LDL-cholesterol (calculated using the Friedewald formula). All these measurements were performed on plasma from fasting individuals using a Cobas 6000 analyzer (Roche diagnostics). Serum creatinine was measured (colorimetry, Kone Optima, Thermolab System, Paris La Défense, France) and creatinine clearance estimated (using the Chronic Kidney Disease-Epidemiology Collaboration equation). The urinary albumin/creatinine ratio (UACR) was measured (laser immunonephelometry, BN100, Dade-Behring, Paris, France).
- Diabetes-related complications: retinopathy (defined as any medical argument for a retinopathy); nephropathy [defined as renal failure (estimated creatinine clearance < 60 mL/min) and/or albuminuria (UACR > 3 mg/mmol)]; neuropathy (defined as any sign or symptoms of polyneuropathy); peripheral arterial occlusive disease (stenosis measured 50% by ultrasound examination); macroangiopathy (defined as peripheral arterial occlusive disease or history of stroke, heart failure or coronary artery disease).

### Statistical analyses

Continuous variables were expressed as means ± standard deviation and compared using one-way ANOVA or the Mann–Whitney’s U test as appropriate. No data replacement procedure was used for missing data. Pearson’s and/or Spearman’s correlations were performed to identify the parameters associated with EAT. The χ^2^ test was used to measure significant differences between the proportion of patients with a CAC score < 100 and those with a score ≥ 100 AU. Logistic regression was performed for the multivariable analysis, which included only those parameters associated with a CAC score ≥ 100 AU, as well as BMI and gender. Odds ratios with 95% confidence intervals (95 CI) for the risk of a CAC score ≥ 100 AU were calculated. Statistical analyses were performed using SPSS software (SPSS, Chicago, IL). The level of significance for all tests was p < 0.05.

## Results

### Patient characteristics

Of the 410 patients who met study inclusion criteria, 1 was not included as the EAT volume could not be measured. The characteristics of the 409 included patients are shown in Table 1.

**Table 1:** Patient characteristics

	Available data	Total
Clinical characteristics		
Age (years)	n = 409	57.1 ± 12.4
Gender (Male/Female)	n = 409	218/191
Body mass index (kg/m^2^)	n = 403	29.1 ± 5.9
Overweight or obese	n = 403	325 (79.5)
Obese	n = 403	158 (38.6)
Ethnicity	n = 408	
Caucasian		88 (21.5)
Sub-Saharan Africa—Antilles		103 (25.2)
Middle East, North Africa		146 (35.7)
Asia		57 (14.0)
Other		14 (3.4)
Diabetes		
Type	n = 409	
Type 1		56 (13.7)
Type 2		318 (77.8)
Other		35 (8.6)
Treatment		
Oral antidiabetic agents	n = 408	295 (72.1)
Glucagon-like peptid 1 agonists	n = 408	75 (18.3)
Insulin	n = 408	252 (61.6)
Time since diagnosis (years)	n = 409	14.0 ± 10.2
HbA1c, %	n = 403	9.0 ± 2.3
Retinopathy	n = 392	160 (39.1)
Macular edema	n = 382	44 (10.8)
Nephropathy	n = 390	148 (36.2)
Albuminuria	n = 389	135 (33.0)
Renal failure	n = 409	39 (9.5)
Neuropathy	n = 401	163 (39.9)
Peripheral arterial occlusive disease	n = 409	37 (9.0)
History of stroke	n = 409	13 (3.2)
History of heart failure	n = 356	5 (1.2)
Coronary artery disease (CAD)	n = 409	4 (1.0)
Additional cardiovascular risk factors		
Family history of premature CAD	n = 149	33 (8.1)
Hypertension	n = 408	224 (54.8)
Systolic blood pressure (mmHg)	n = 408	135 ± 18
Diastolic blood pressure (mmHg)	n = 408	77 ± 13
Dyslipidemia	n = 408	238 (58.2)
Total cholesterol (mmol/L)	n = 409	4.4 ± 1.2
HDL cholesterol (mmol/L)	n = 409	1.2 ± 0.4
Triglycerides (mmol/L)	n = 409	1.7 ± 1.2
LDL cholesterol (mmol/L)	n = 406	2.4 ± 0.9
Non HDL cholesterol (mmol/L)	n = 409	3.2 ± 1.1
Current smoking	n = 409	71 (17.4)
Smoking history (pack-year)	n = 386	8.2 ± 16.6
Computed tomography results		
Epicardial adipose tissue volume (cm^3^)	n = 409	92 ± 37
CAC score (Agatston unit)	n = 409	116 ± 332

Data are n (%) or mean ± standard deviation

*CAC* coronary artery calcium score, *CAD* coronary artery disease

### Parameters associated with EAT

**Fig. 1:**
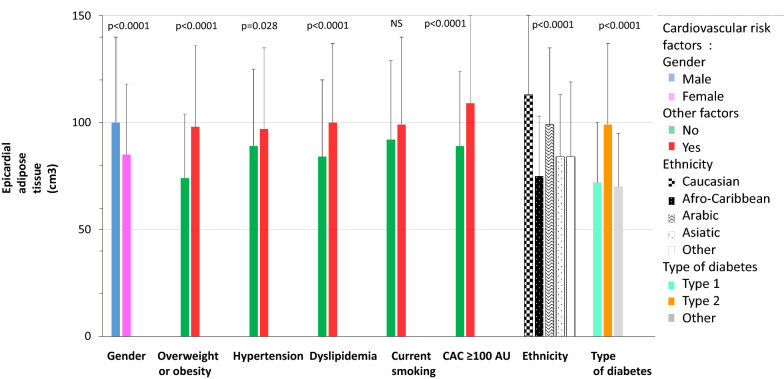
Epicardial adipose tissue volume according to cardio-vascular risk factors. Data are mean ± SD. *AU* Agatston unit, *CAC* coronary artery calcification score

**Fig. 2:**
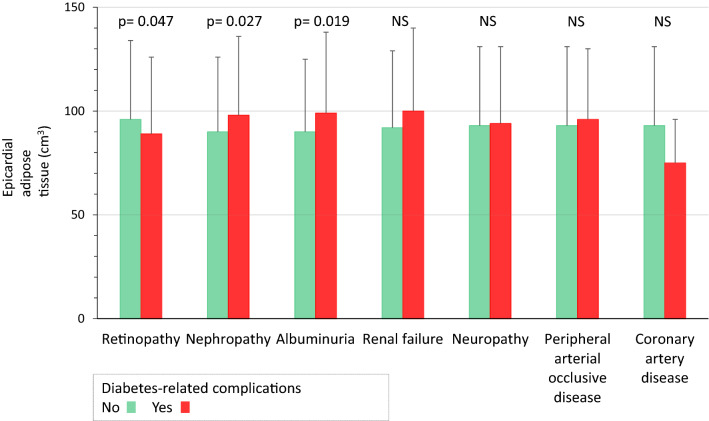
Epicardial adipose tissue volume according to diabetes-related complications. Data are mean ± SD

EAT volume was positively associated with age, BMI, triglyceride levels, cumulative tobacco consumption and CAC score. It was negatively correlated with creatinine clearance and HDL-cholesterol level (Table 2). EAT volume was higher in males, overweight and obese persons, those categorized as Caucasian, patients with T2D, those with hypertension, individuals with dyslipidaemia and people with a CAC score ≥ 100 AU (Fig. 1, Additional file 1: Table S1). It was also higher in participants with nephropathy and albuminuria, but lower in patients with retinopathy (Fig. 2, Additional file 1: Table S2).

**Table 2:** Correlation of epicardial adipose tissue volume with quantitative data

	R	p-value
Age, years	0.324	< 0.0001
Body mass index, kg/m^2^	0.288	< 0.0001
HbA1c, %	− 0.093	0.142
Urinary albumin/creatinin ratio, mg/mmol	0.059	0.260
Creatinine clearance, mL/min	− 0.237	< 0.0001
Systolic blood pressure, mmHg	− 0.022	0.656
Diastolic blood pressure, mmHg	− 0.035	0.483
Total cholesterol, mmol/L	− 0.057	0.250
HDL cholesterol, mmol/L	− 0.190	< 0.0001
Triglycerides, mmol/L	0.150	0.002
LDL cholesterol, mmol/L	− 0.070	0.158
Non HDL cholesterol, mmol/L	0.005	0.925
Smoking history (pack-year)	0.233	< 0.0001
Coronary artery calcium score, Agatston unit	0.287	< 0.0001

Additional file 1: Table S3 also shows that the use of the following treatments was associated with a higher EAT volume: metformin, sulfonylurea, glucagon-like peptide 1 receptor agonists, beta-blockers, statins and aspirin.

### Parameters associated with CAC

Individuals with a CAC score ≥ 100 AU (versus < 100 AU) were older, more likely to be Caucasian (versus Afro-Caribbean, Arabic and Asian), more likely to have T2D (versus T1D), and more likely to have retinopathy, macular edema, nephropathy, macrovascular disease, hypertension and dyslipidaemia. Furthermore, they had had diabetes for a longer time and had higher pack-year smoking history (Table 3). In the multivariable analysis, after adjustment for all these parameters and both gender and BMI, EAT was independently associated with a CAC score ≥ 100 AU (Table 4).

**Table 3:** Patient characteristics according to coronary artery calcium scores < or ≥ 100 Agatston units

	CAC < 100 AU	CAC ≥ 100 AU	OR [95%CI]	p
	n = 320	n = 89		
Clinical characteristics				
Age (years)	54.7 ± 12.2	65.7 ± 8.7		< 0.0001
Gender (Male/Female)	164/156	54/35	0.7 [0.4–1.1]	0.120
Body mass index (kg/m^2^)	29.3 ± 6.0	28.2 ± 5.5		0.119
Ethnicity				
Caucasian	57(17.9)	31(34.8)	REF	
Afro-Caribbean	88(27.6)	15(16.9)	0.3 [0.2–0.6]	0.001
Arabic	116(36.4)	30(33.7)	0.5 [0.3–0.9]	0.014
Asia	47(14.7)	10(11.2)	0.4 [0.2–0.9]	0.023
Other	11(3.4)	3(3.4)	0.5 [0.1–1.9]	0.316
Diabetes				
Type				
Type 1	52(16.2)	4(4.5)	REF	
Type 2	239(74.7)	79(88.8)	4.3 [1.5–12.3]	0.006
Other	29(9.1)	6(6.7)	2.7 [0.7–10.3]	0.149
Time since diagnosis (years)	12.9 ± 9.8	17.7 ± 10.8		< 0.0001
HbA1c (%)	9.0 ± 2.3	8.7 ± 2.1		0.280
Diabetes-related complication				
Retinopathy	116(37.8)	44(51.8)	1.8[1.1–2.9]	0.025
Macular edema	28(9.3)	16(19.5)	2.4 [1.2–4.6]	0.018
Nephropathy	100(33.0)	48(55.2)	2.5 [1.5–4.1]	< 0.0001
Neuropathy	120(38.5)	43(48.3)	1.5 [0.9–2.4]	0.112
Macrovascular disease	30(9.4)	32(36.0)	5.4 [3.1–9.6]	< 0.0001
Additional cardiovascular risk factors				
Family history of premature CAD	27(21.8)	6(24.0)	1.1 [0.4–3.1]	0.795
Hypertension	163(50.9)	61(69.3)	2.2 [1.3–3.6]	0.002
Dyslipidaemia	171(53.4)	67(76.1)	2.8 [1.6–4.8]	< 0.0001
Current smoking	52(16.2)	19(21.3)	1.4 [0.8–2.5]	0.270
Cumulative tobacco consumption (pack-year)	6.4 ± 14.1	14.7 ± 22.8		< 0.0001

Data: mean ± standard deviation

*AU* Agatston unit, *CAD* coronary artery disease, *OR* odds ratio, *95% CI* 95% confidence interval

**Table 4:** Parameters explaining a coronary artery calcium score ≥ 100 Agaston units in multivariable analysis

	Odds ratio	95% confidence interval	p-value
Epicardial adipose tissue (per 10 cm^3^)	1.13	1.04–1.23	0.004
Age (per year)	1.08	1.05–1.12	< 0.001
Body mass index (per kg/m^2^)	0.94	0.89–1.00	0.051
Male vs Female			NS
Type 2 vs Type 1 diabetes			NS
Other diabetes types vs Type 1 diabetes			NS
Afro-Caribbean vs Caucasian			NS
Arabic vs Caucasian			NS
Asia vs Caucasian			NS
Other vs Caucasian			NS
Hypertension			NS
Dyslipidaemia			NS
Diabetes duration			NS
Cumulative Tobacco consumption (per pack-year)	1.03	1.01–1.04	0.002
Retinopathy	1.89	0.99–3.58	0.05
Macular edema			NS
Nephropathy			NS
Macrovascular disease	3.94	1.92–8.07	< 0.001

## Discussion

Our cohort study results show that EAT volume was independently associated with CAC in people with diabetes. This reflects results from another study with a mix of individuals with and without diabetes [16]. Similarly, Yerramasu et al. found that EAT volume was an independent marker of both the presence and severity of CAC burden in 333 asymptomatic patients with T2D [11]. However, the latter finding goes against results from three others studies where no such association was found [12–14]. There are several possible reasons for this. First, these three studies had less statistical power than Yerramasu’s and ours, as they only included between 95 and 200 patients [12–14]. Second, one of the three (Christensen et al.) measured EAT thickness not volume [14]. Third, inclusion criteria differed between the three studies: only patients with T2D and elevated urinary albumin excretion rate were recruited in Christensen et al.’s study [14], while only young Native Americans with T2D were included in Reinhardt et al.’s study [13]. Our results in diabetic persons are clinically relevant as we were able to show that the association remained significant even after adjustment for numerous confounding factors.

### Parameters associated with EAT volume, to be considered as confounders

First, EAT volume was positively correlated with male gender and older age. Elsewhere, EAT volume has been associated with all the components of metabolic syndrome in people with T2D [16, 17] and those with T1D [18, 19]. Similarly, we found an association between EAT volume and higher BMI, increased triglycerides levels, lower HDL-cholesterol levels and antihypertensive treatment. This result is in line with previous studies which showed a higher EAT volume in individuals with T2D than those with T1D [12, 19]. Additionally, cumulative tobacco consumption, which is associated with insulin resistance and metabolic syndrome [20], was positively correlated with EAT volume in our study. Finally, we also found that EAT volume was higher in individuals of Caucasian ethnicity. Similarly, the difference in EAT thickness between persons with and without metabolic syndrome was more evident in Caucasians [17, 21]. Other studies have also found that EAT levels differ according to racial/ethnic group in the general population [22–25].

### EAT and subclinical atherosclerosis, including CAC

The association between EAT and CAC in our study suggests that EAT might play a role in subclinical atherosclerosis in diabetes. There are arguments to support this hypothesis. First, increased EAT volume/thickness has been associated with markers of subclinical atherosclerosis other than CAC score, such as coronary artery disease and cardiac dysfunction [5]. Second, prospective studies have shown that high EAT volume/thickness is associated with more cardiovascular events in the general population [26], in patients with T2D without participant-selection study criteria [27] and T2D patients with microalbuminuria [14]. Third, EAT identifies individuals at increased risk of CAC progression [11]. Fourth, whereas EAT is physiologically cardioprotective—as it provides mechanical protection and energy to the myocardium and has anti-inflammatory properties—, abnormally increases in EAT volume are proinflammatory [3–6]. Furthermore, EAT secretes vasoactive factors that regulate coronary endothelial function and facilitate free fatty acid influx [3–6]. Therefore, the positive association between EAT and CAC may reflect early pathophysiological effects of EAT on local atherosclerosis. EAT volume could therefore be considered as a surrogate of coronary atherosclerosis. Similarly, EAT volume has been associated with plaque vulnerability, which may contribute to acute coronary syndrome [28]. Finally, EAT might in addition directly promote calcification processes in atheroma [29].

### EAT and diabetic microangiopathic complications

We found that EAT volume was positively associated with albuminuria—without correlation with renal failure—whereas such a correlation was not found previously in the diabetic population [11, 12]. Albuminuria is a known marker of poor cardiovascular prognosis in people with diabetes [8, 10]. We might hypothesize that the association between EAT and albuminuria only reflects EAT-induced coronary endothelial dysfunction through paracrine effects [30]. On the contrary, EAT volume was lower in case of retinopathy. In the study by Yerramasu et al., the percentage of retinopathy also decreased by increasing EAT tertiles (34, 27 and 23%, respectively) but statistical significance was not reached (p = 0.18) [11]. Whether EAT would play a systemic role on eyes need further specific studies. Finally, as previously reported [11], EAT volume did not differ by presence of neuropathy.

### Clinical and therapeutic implications

Our results suggest that measuring EAT volume might improve assessment of cardiovascular prognosis, but whether the prognostic value of EAT is additional to the one of CAC score is unknown [14, 26, 27]. There are also therapeutic implications of our work. EAT volume can be modified by lifestyle such as diet and/or exercise, bariatric surgery and pharmaceutical interventions [31, 32]. For example, thiazolidinediones, dipeptidyl peptidase-4 inhibitors, glucagon-like peptide-1 receptor agonists and sodium-glucose cotransporter 2 inhibitors have all been reported to reduce EAT volume [33]. On the contrary, CAC cannot regress.

### Limitations and strengths of the study

Our study has several limitations. Its design was observational, which prevented us from being able to draw conclusions about causal relationships between EAT volume and CAC. Neither were we able to make a conclusion about the role of therapy on EAT volume. Furthermore, in order to evaluate the prognostic value of EAT volume, we used a marker of subclinical atherosclerosis (i.e., CAC) instead of measuring the incidence of cardiovascular events. Moreover, we only included patients who had been admitted to our hospital department and who had their CAC score measured. Therefore, our results may not be representative of all patients with diabetes. Finally, we did not have data on waist circumference, which could have influenced the association between EAT volume and CAC. However, we did adjust for several confounders, including BMI.

The main strength of our study is that we measured EAT and not pericardial (or total cardiac) adipose tissue. EAT lies between the myocardium and the visceral layer of the pericardium and is different from pericardial fat which is located externally to the myocardium. There is no fascia separating EAT and myocardium. Therefore both tissues are in direct contact [3–6, 31]. To date, EAT is the only type of cardiac adipose tissue which has been observed to predict incident cardiovascular events in T2D patients [27]. Furthermore, we applied a robust methodology—CT acquisition and assessment following standard methods—as well as cardiac software to automatically quantify EAT. Only 1 EAT measure out of the 410 performed (study population) could not be interpreted. CT scans are considered the gold standard for EAT as, unlike echography, they measure EAT volume not thickness [4, 14].

## Conclusions

We showed that in individuals with diabetes, EAT volume was higher in males, persons of Caucasian ethnicity and those who met the criteria for the components of metabolic syndrome. More importantly, we demonstrated that EAT was independently associated with subclinical coronary atherosclerosis in our study population. This suggests that EAT quantification might improve early assessment of cardiovascular prognosis. Furthermore, the anatomic proximity of EAT and adjacent myocardial and vascular tissues might provide an opportunity for therapeutic interventions.

## Supplementary Information

**Additional file 1: Table S1.** Epicardial adipose tissue volume according to cardio-vascular risk factors. **Table S2.** Epicardial adipose tissue volume according to diabetes-related complications. **Table S3.** Epicardial adipose tissue volume according to treatment.

## Data Availability

Data for the present analysis can be provided from the first author on reasonable request.

## Abbreviations

AU: Agatston unit
BMI: Body mass index
CAC: Coronary artery calcium
CT: Computed tomography
EAT: Epicardial adipose tissue
PACS: Picture Archiving and Communication Systems
T1D: Type 1 diabetes
T2D: Type 2 diabetes
UACR: Urinary albumin/creatinine ratio
