# Supplementary material for: Epicardial adipose tissue volume and coronary calcification among people living with diabetes: a cross-sectional study
Source: Cardiovasc Diabetol. 2021 Feb 5;20:35. doi: 10.1186/s12933-021-01225-6 (PMC7863354; doi:10.1186/s12933-021-01225-6)
Supplement: Supplementary file 1 — Additional file 1: Table S1. Epicardial adipose tissue volume according to cardio-vascular risk factors. Table S2. Epicardial adipose tissue volume according to diabetes-related complications. Table S3. Epicardial adipose tissue volume according to treatment. [file 12933_2021_1225_MOESM1_ESM.docx]

**Additional Table S1: Epicardial adipose tissue volume according to cardio-vascular risk factors**

|  | Available data | Epicardial adipose tissue (cm^3^) | P-value |
| --- | --- | --- | --- |
| Gender (Male/Female) |  |  | <0.0001 |
| Male | n = 218 | 100±40 |  |
| Female | n = 191 | 85±33 |  |
| Overweight or obesity |  |  | <0.0001 |
| No | n = 78 | 74±30 |  |
| Yes | n = 325 | 98±38 |  |
| Ethnicity |  |  | <0.0001 |
| Caucasian | n = 88 | 113±44 |  |
| Afro-Caribbean | n = 103 | 75±28 |  |
| Arabic | n = 146 | 99±36 |  |
| Asia | n = 57 | 84±29 |  |
| Other | n = 14 | 84±35 |  |
| Type of diabetes |  |  | <0.0001 |
| Type 1 diabetes | n = 56 | 72±28 |  |
| Type 2 diabetes | n = 318 | 99±38 |  |
| Other types of diabetes | n = 35 | 70±25 |  |
| Family history of premature CAD |  |  | 0.480 |
| No | n = 116 | 91±36 |  |
| Yes | n = 33 | 96±43 |  |
| Hypertension |  |  | 0.028 |
| No | n = 184 | 89±36 |  |
| Yes | n = 224 | 97±38 |  |
| Dyslipidaemia |  |  | <0.0001 |
| No | n = 170 | 84±36 |  |
| Yes | n = 238 | 100±37 |  |
| Current smoking |  |  | 0.125 |
| No | n = 338 | 92±37 |  |
| Yes | n = 71 | 99±41 |  |
| CAC ≥ 100 AU |  |  | <0.0001 |
| No | n = 320 | 89±35 |  |
| Yes | n = 89 | 109±41 |  |

AU: Agatston unit; CAC: coronary artery calcification score; CAD: coronary artery disease

Data: mean ± standard deviation

Additional **Table S2: Epicardial adipose tissue volume according to diabetes-related complications**

|  | Available data | Epicardial adipose tissue (cm^3^) | P-value |
| --- | --- | --- | --- |
| Retinopathy |  |  | 0.047 |
| No | n = 232 | 96±38 |  |
| Yes | n = 160 | 89±37 |  |
| Macular edema |  |  | 0.669 |
| No | n = 338 | 94±38 |  |
| Yes | n = 44 | 91±36 |  |
| Nephropathy |  |  | 0.027 |
| No | n = 242 | 90±36 |  |
| Yes | n = 148 | 98±38 |  |
| Albuminuria |  |  | 0.019 |
| No | n = 254 | 90±35 |  |
| Yes | n = 135 | 99±39 |  |
| Renal failure |  |  | 0.246 |
| No | n = 370 | 92±37 |  |
| Yes | n = 39 | 100±40 |  |
| Neuropathy |  |  | 0.892 |
| No | n = 238 | 93±38 |  |
| Yes | n = 163 | 94±37 |  |
| Peripheral arterial occlusive disease |  |  | 0.606 |
| No | n = 372 | 93±38 |  |
| Yes | n = 37 | 96±34 |  |
| History of stroke |  |  | 0.691 |
| No | n = 396 | 93±37 |  |
| Yes | n = 13 | 97±41 |  |
| History of heart failure |  |  | 0.397 |
| No | n = 48 | 94±30 |  |
| Yes | n = 5 | 107±51 |  |
| Coronary artery disease |  |  | 0.331 |
| No | n = 405 | 93±38 |  |
| Yes | n = 4 | 75±21 |  |
| Macrovascular disease |  |  | 0.733 |
| No | n = 347 | 93±38 |  |
| Yes | n = 62 | 95±38 |  |

Data: mean ± standard deviation

**Additional Table S3: Epicardial adipose tissue volume according to treatment**

|  | N | Mean ± SD | P-value |
| --- | --- | --- | --- |
| Metformin |  |  | 0.003 |
| No | n = 138 | 86±35 |  |
| Yes | n = 270 | 97±38 |  |
| Sulfonylurea |  |  | 0.044 |
| No | n = 252 | 90±38 |  |
| Yes | n = 156 | 98±36 |  |
| Alpha-glucosidase inhibitor |  |  | 0.719 |
| No | n = 403 | 93±38 |  |
| Yes | n = 5 | 87±15 |  |
| Di-peptidyl-peptidase 4 inhibitor |  |  | 0.908 |
| No | n = 302 | 93±38 |  |
| Yes | n = 106 | 93±36 |  |
| Sodium-glucose cotransporter-2 inhibitor |  |  | 0.349 |
| No | n = 406 | 93±38 |  |
| Yes | n = 2 | 68±21 |  |
| Oral hypoglycaemic agent |  |  | <0.0001 |
| No | n = 113 | 81±32 |  |
| Yes | n = 295 | 98±39 |  |
| Glucagon-like peptide 1 receptor agonists |  |  | 0.007 |
| No | n = 333 | 91±37 |  |
| Yes | n = 75 | 104±38 |  |
| Insulin |  |  | 0.529 |
| No | n = 156 | 95±37 |  |
| Yes | n = 252 | 92±38 |  |
| Angiotensin-converting enzyme inhibitor |  |  | 0.290 |
| No | n = 319 | 92±38 |  |
| Yes | n = 89 | 97±36 |  |
| Angiotensin II Receptor Blocker |  |  | 0.234 |
| No | n = 298 | 92±37 |  |
| Yes | n = 110 | 97±40 |  |
| Beta blocker |  |  | 0.007 |
| No | n = 356 | 91±37 |  |
| Yes | n = 52 | 106±39 |  |
| Calcium channel inhibitor |  |  | 0.161 |
| No | n = 312 | 92±37 |  |
| Yes | n = 96 | 98±39 |  |
| Hydrochlorothiazide |  |  | 0.418 |
| No | n = 395 | 93±38 |  |
| Yes | n = 13 | 102±37 |  |
| Spironolactone |  |  | 0.851 |
| No | n = 403 | 93±38 |  |
| Yes | n = 5 | 96±14 |  |
| Statin |  |  | 0.000 |
| No | n = 194 | 86±36 |  |
| Yes | n = 214 | 100±38 |  |
| Fibrates |  |  | 0.107 |
| No | n = 399 | 93±37 |  |
| Yes | n = 9 | 113±42 |  |
| Ezetimibe |  |  | 0.297 |
| No | n = 397 | 93±38 |  |
| Yes | n = 11 | 105±25 |  |
| Aspirin |  |  | 0.002 |
| No | n = 310 | 90±37 |  |
| Yes | n = 98 | 104±39 |  |

Data: mean ± standard deviation
